# Picosecond Laser Interference Patterning of Periodical Micro-Architectures on Metallic Molds for Hot Embossing

**DOI:** 10.3390/ma12203409

**Published:** 2019-10-18

**Authors:** Yangxi Fu, Marcos Soldera, Wei Wang, Bogdan Voisiat, Andrés Fabián Lasagni

**Affiliations:** 1 Institut für Fertigungstechnik, Technische Universität Dresden, George-Bähr-Str. 3c, 01069 Dresden, Germany; marcos.soldera@mailbox.tu-dresden.de (M.S.); wei.wang2@mailbox.tu-dresden.de (W.W.); bogdan.voisiat@tu-dresden.de (B.V.); andres_fabian.lasagni@tu-dresden.de (A.F.L.); 2 PROBIEN-CONICET, Dto. de Electrotecnia, Universidad Nacional del Comahue, Buenos Aires 1400, Neuquén 8300, Argentina; 3 Fraunhofer-Institut für Werkstoff-und Strahltechnik IWS, Winterbergstr. 28, 01277 Dresden, Germany

**Keywords:** direct laser interference patterning, microstructures, laser-induced periodic surface structures, hot embossing, polymethyl methacrylate

## Abstract

In this work, it is demonstrated that direct laser interference patterning (DLIP) is a method capable of producing microtextured metallic molds for hot embossing processes. Three different metals (Cr, Ni, and Cu), relevant for the mold production used in nanoimprinting systems, are patterned by DLIP using a picosecond laser source emitting at a 532 nm wavelength. The results show that the quality and surface topography of the produced hole-like micropatterns are determined by the laser processing parameters, such as irradiated energy density and the number of pulses. Laser-induced periodic surface structures (LIPSS) are also observed on the treated surfaces, whose shapes, periodicities, and orientations are strongly dependent on the accumulated fluence. Finally, the three structured metals are used as embossing molds to imprint microlenses on polymethyl methacrylate (PMMA) foils using an electrohydraulic press. Topographical profiles demonstrate that the obtained structures are comparable to the masters showing a satisfactory reproduction of the texture. The polymeric microlens arrays that showed the best surface homogeneity and overall quality were those embossed with the Cr molds.

## 1. Introduction

In recent decades, it has been shown that surfaces with deterministic periodic topographies exhibit enhanced surface properties compared to their flat counterpart [1]. For instance, many studies have shown that micro- and nano-structures have a profound impact on reflected colors [2,3], tribological behavior [4], wettability properties [5,6], and initial bacterial adhesion [7]. 

To date, various transferring techniques have been utilized for structure microfabrication in order to meet a variety of application needs, including inject molding, thermoforming, soft lithography, UV nanoimprinting, and hot embossing. For all these transferring techniques, mold fabrication plays a crucial role in order to achieve high quality and high precision of the replicated patterns [8,9,10,11].

Among the above mentioned replication methods, hot embossing is regarded as one of the most promising industrial-scalable processes for producing high-precision and high-quality structures at the micro- and nano-scale. It makes use of the difference in the thermomechanical properties of a hard master mold and a polymeric substrate for parallel replication of structures from the master to the polymer [8,12]. Generally, masters used in hot embossing can be made of silicon [13,14], metals [15], photoresists [16], or epoxy resins [17]. Among these, metal molds present significant advantages, since they can be used at higher pressures and temperatures and retain their structures after a large number of cycles. Additionally, metals also have the potential to be used in large-area seamless sleeves for roll-to-roll hot embossing [18,19]. The most frequently used methods to fabricate the metal master are micromachining, electroplating, etching, photolithography, and laser structuring, among others [8,12,15]. Despite these methods are well-established, they still possess some disadvantages, such as high cost, significant time consumption, limitation of feature resolution, and inaccurate dimension control [8,16,20].

Direct laser interference patterning (DLIP), a cost-effective one-step technique for texturing periodic microstructures with high resolution and throughput on a variety of materials [21,22,23], including metals [24,25,26,27], polymers [28,29] and ceramics [30,31], provides an efficient approach for molds patterning not only for hot embossing systems, but also for injection molding and UV nanoimprinting. In this method, an interference pattern generated by two or more coherently overlapping laser beams on the sample surface is employed to directly ablate the material at the intensity maxima positions producing periodic structures on its surface. Due to the interference phenomenon, a large number of periodic microelements can be generated with a single laser pulse, which yields outstanding processing speeds approaching 1 m^2^/min [32,33]. Depending on the laser processing parameters, such as pulse duration, laser wavelength, polarization, energy density, and interference angle, the DLIP method allows the fabrication of periodic microstructure arrays in the micrometer and sub-micrometer scale with controlled topographical parameters (shape, period, and depth) [33,34,35]. The most stringent requirements of DLIP are that the material to be treated must absorb the laser radiation at the selected wavelength and sufficient pulse energy must be provided to ablate the material directly [36,37,38]. Generally, polymers and ceramics are processed with UV laser radiation, whereas metals are treated with visible and near-infrared wavelengths [38]. 

Nickel is one of the most widely used materials for hot embossing molds due to its remarkable durability, ease of cleaning, lack of oxidation, and compatibility with many polymers during the hot embossing process [39,40,41,42]. However, nickel may cause skin contact allergies in sensitive people, and large doses of nickel exposure also pose a threat to human health [43,44,45]. It has already been demonstrated that two-beam DLIP is capable of patterning “line-like” microstructures on seamless Ni sleeves, which were successfully used as cylindrical molds for hot embossing polyethylene terephthalate (PET) foils in a roll-to-roll system, reaching a maximum throughput of 15 m^2^/min [18].

The aim of this contribution is to further optimize the microstructuring process of metallic molds using four-beam laser interference patterning to produce “hole-like” periodic arrays with higher surface-area-to-volume ratios than the simpler “line-like” grooves achieved by two-beam DLIP. Another objective of this work is to find a replacement for Ni molds to improve worker health and safety. To this end, a comparison study of surface periodic micropatterns produced by picosecond DLIP on nickel and two potential candidates for metal molds, namely chromium and copper, is performed in this work. Hole-like periodic patterns with a spatial period of 4.7 µm were produced by overlapping four laser beams. Under identical laser processing parameters, the obtained surface patterns on those metals are characterized and compared. Finally, the three studied structured metals were used as embossing molds to imprint micropatterns on polymethyl methacrylate (PMMA) foils using an electrohydraulic press.

## 2. Materials and Methods

### 2.1. Materials

Three different metals, i.e., chromium (Cr), nickel (Ni), and copper (Cu) were used in the experiments, which were provided by Sächsische Walzengravur GmbH. In order to investigate the effect of the surface roughness, the arithmetic mean roughness ($R_{a}$) of untreated metal sheets was measured using a confocal microscope. The untreated nickel and chromium samples have an average surface roughness of $R_{a}$ = 0.05 µm, while the copper sample has a higher roughness of $R_{a}$ = 0.17 µm. Thickness and roughness of the metal samples together with some of their physical properties are presented in Table 1. Before the laser process, the substrates were thoroughly cleaned with 2-propanol in an ultrasonic bath at room temperature to remove surface contaminants. The polymeric substrates used in the hot embossing process are 200 µm thick polymethyl methacrylate (PMMA) foils (PLEXIGLAS@, Evonik Performance Materials GmbH, Weiterstadt, Germany). Their glass transition temperature is 109 °C according to the manufacturer.

### 2.2. DLIP Experimental Details

A self-developed interference setup (VIS/IR-DLIP µFAB, Fraunhofer IWS, TU Dresden, Dresden, Germany) was utilized to produce periodic hole-like patterns by overlapping four Gaussian beams (TEM00 mode) on the metal sheet surfaces. The laser source is a picosecond-pulsed laser system (NeoLASE GmbH, Hannover, Germany) with a wavelength of 532 nm, a pulse duration of 70 ps and a repetition rate up to 10 kHz. 

**Figure 1:**
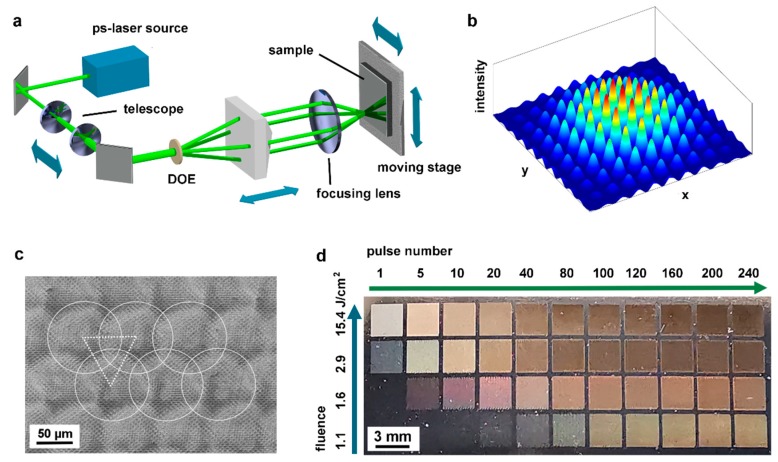
(**a**) Schematic of the DLIP experimental setup. (**b**) Intensity distribution of four symmetrically-distributed beams’ interference. (**c**) SEM image of microstructures fabricated by DLIP on Cr (fluence: 1.6 J/cm^2^, 10 pulses). White circles indicate the laser interference spots irradiated on the Cr surface, which are approximately 102 µm in diameter and arranged in a triangular pattern (white dot line) with an overlap of 30%. (**d**) Photograph of a four-beam laser processed Cr sheet. The laser fluence was increased from 1.1 J/cm^2^ to 15.4 J/cm^2^ (from the bottom to the top) and the pulse number was raised from 1 to 240 (from left to right).

To produce the hole-like interference pattern, the laser beam is split into four sub-beams with the same intensity using a diffractive optical element (DOE), as shown schematically in Figure 1a. The sub-beams are then parallelized through a pyramidal prism and overlapped onto the sample surface using a lens. Figure 1b shows schematically the intensity distribution for this four-beam interference patterning resembling a 2D sinusoidal function modulated by a Gaussian function. By changing the pyramid position (in respect to the pyramid), the angle between the overlapping beams can be varied, and therefore the spatial period can be adjusted. For all the experiments carried out in this work, a fixed spatial period of 4.7 µm was used. The spot size on the surface was varied from 48 µm to 147 µm by adjusting the distance between the lenses of the microscope which, in turn, yielded a variation of the laser fluence from 1.1 J/cm^2^ to 15.4 J/cm^2^ (see Figure 1c). During the process, the sample was mounted on an XY moving stage (travel distance 200 mm, max. speed 300 m/s, resolution 0.1 µm, Aerotech GmbH, Nürnberg, Germany), which was used to position the sample in respect to the laser beam.

### 2.3. Plate-to-Plate Hot Embossing

The three laser structured metals, i.e., Cr, Ni, and Cu, were used as embossing molds to transfer the micropatterns on polymethyl methacrylate (PMMA) foils using an electrohydraulic press (Paul-Otto Weber GmbH, Remshalden, Germany). PMMA is classified as a thermoplastic polymer, which can be molded in a large temperature range beginning at the elastic range and ending in the stage of melting [8,50,51]. The preset imprint temperature was 100 °C but it varied during the hot embossing process between 95 °C and 105 °C. These temperatures are lower than the PMMA glass transition temperature to prevent the polymer from sticking onto the master tightly and protecting the transferred texture from damage and breakage during demolding. A relatively high compression force of 200 kN was applied to force the polymer to enter the viscoplastic deformation regime. An embossing time of 5 min is applied to ensure the soften PMMA completely fills the stamp cavities. No additional anti-sticking coating was deposited on the metal molds prior to embossing. 

### 2.4. Characterization Methods

After the laser treatment, a high-resolution scanning electronic microscopy (Philips XL30 ESEM-FEG, Hillsboro, OR, USA) with a voltage of 10 kV was utilized to visualize the patterned surface. Surface topography of molds and imprinted polymers was determined using a confocal optical profiler (Sensofar S neox, Schaefer Technologie GmbH, Langen, Germany) with an 150× objective, which provides a lateral and vertical resolution of 140 nm and 1 nm, respectively. The measured 3D profiles were processed by SensoMap software (SensoMap „Premium“ Version 7, Schaefer Technologie GmbH, Langen, Germany) to calculate the structure depth and periodicity of the patterns.

## 3. Results and Discussion

### 3.1. Patterning Strategy

The Ni, Cr, and Cu surfaces are irradiated with laser spots forming a compact and regular triangular array, in order to achieve homogeneous structures over large areas. A given number of laser pulses is irradiated in each spot before positioning the sample on the adjacent spot. For instance, the scanning electron microscope (SEM) image of Figure 1c shows a regular ordered hole-like pattern with a spatial period of Λ = 4.7 µm on a Cr surface, irradiated with 10 pulses per spot at a laser fluence of 1.6 J/cm^2^. The white circles in the image indicate the laser interference spots with a diameter of 102 µm. Due to the Gaussian distribution of the laser beam intensity, the structure depth decreases close to the circle edges and therefore adjacent spots have to be overlapped to obtain a homogeneous texture on large areas. It was found that the optimum pulse-to-pulse separation is 70 µm representing an approximate 30% overlap. 

Following this patterning strategy, matrices containing structured areas (3 × 3 mm^2^ in size) were produced on each studied metal varying the number of laser pulses and fluence in order to optimize the texture homogeneity and achieve the maximum possible structure depth. Figure 1d shows an optical photograph of the DLIP-treated Cr substrate. The blue arrow in the image indicates the increase in laser fluence per pulse from 1.1 J/cm^2^ to 15.4 J/cm^2^, and the green arrow shows the pulse number increasing from 1 to 240. It can be seen that the appearance of the laser processed area changes from the shiny untreated metal (bottom left corner) to a matte and dark finish (top right corner). This can be associated with the formation of an oxide layer during the laser process and light scattering and diffraction effects caused by the periodic structures on Cr surface [52]. Furthermore, the areas treated with different laser parameters exhibit different reflected colors, attributed to variations in lateral size and structure heights of the induced structures as well as variation in the oxide layer thickness [53]. 

### 3.2. Effect of Laser Fluence

**Figure 2:**
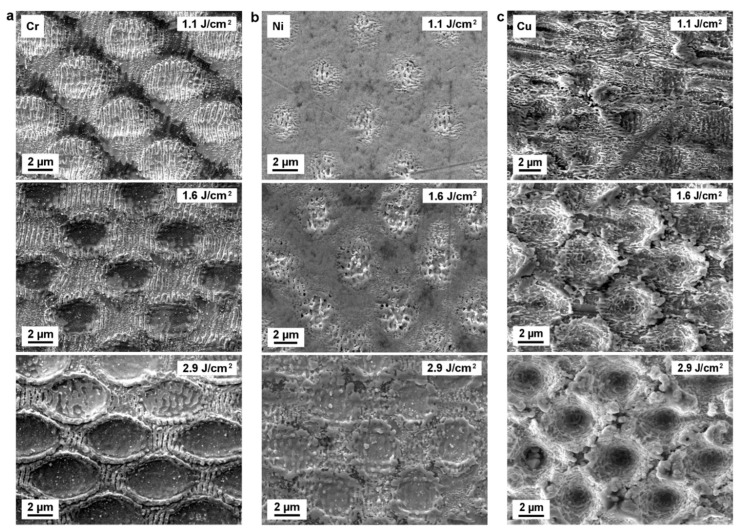
SEM images of (**a**) Cr, (**b**) Ni, and (**c**) Cu structured surfaces using DLIP with 10 (Cr, Ni) or 80 (Cu) laser pulses and different laser fluences of 1.1 J/cm^2^, 1.6 J/cm^2^, and 2.9 J/cm^2^.

Laser fluence, or energy density, is defined as the laser energy deposited per unit of the irradiated area, and it is one of the most important laser processing parameters that control the morphology and quality of the periodic patterns [54]. The SEM images in Figure 2 show periodic microstructures generated by DLIP on Cr (left column), Ni (middle column), and Cu (right column) surfaces irradiated with a fluence of 1.1 J/cm^2^ (top row), 1.6 J/cm^2^ (middle row), and 2.9 J/cm^2^ (bottom row), respectively. For Cr and Ni, 10 pulses per spot were applied. In the case of the Cu substrates, 80 pulses were applied in order to produce a significant surface modification.

As shown in Figure 2a, the desired hole-like pattern on the Cr surface is not recognizable when a low laser fluence of 1.1 J/cm^2^ is used. However, regularly distributed nanoripples (with a period of 0.41 µm) were produced at the interference maxima positions of the irradiated spots. These nanoripples are commonly known as laser-induced periodic surface structures (LIPSS), which will be discussed in detail in Section 3.4. As the irradiated fluence is increased to 1.6 J/cm^2^, the formation of periodic craters becomes visible and LIPSS began to grow also at the interference intensity minima positions. When the laser fluence is further increased to 2.9 J/cm^2^, a larger amount of material is ablated at the areas corresponding to the interference maxima leaving behind craters with a larger depth. The rims of the craters become smooth and slightly raised above the surface. At this fluence, the nanoripples are completely removed from the craters, and they are found between the regularly distributed craters (interference minima positions). The shape of these periodically arranged craters is not circular but elliptical, with an eccentricity of 0.81, due to a subtle misalignment between the prism and converging lens that disturbs the symmetry of the interfering beams.

In Figure 2b, no craters are visible on Ni until the laser fluence was increased to 2.9 J/cm^2^. Similar to chromium laser treated samples, also periodic nanoscaled ripple structures are mainly formed at the intensity maxima of the interference pattern at fluences of 1.1 J/cm^2^ and 1.6 J/cm^2^, however, showing a lower uniformity. As the fluence increases to 2.9 J/cm^2^, circular-shaped craters can be observed on the Ni surface, which have a less defined shape and smaller diameter compared to those on the Cr surface. 

In Figure 2c, the results obtained on Cu are exemplarily shown. In this case, small craters with depths comparable to the original surface roughness are visible at a fluence of 1.1 J/cm^2^. Additionally, some additional features can be distinguished, however, in this case they are not well-defined and their orientation is perpendicular to the LIPSS observed in Ni and Cr. When the laser fluence is risen to 1.6 J/cm², the depth of the craters increases making them more evident. However, the previously visible ripples vanished. In addition, a new type of ripples is visible at the center of the craters. At fluences above 1.6 J/cm^2^, the surface at interference maxima positions is even more molten and ablated yielding regularly distributed and much deeper hole-like structures. The crater rims are wrinkled and do not merge uniformly with adjacent rims. The above-mentioned laser-generated nanoripples can be still found inside almost every hole. 

**Figure 3:**
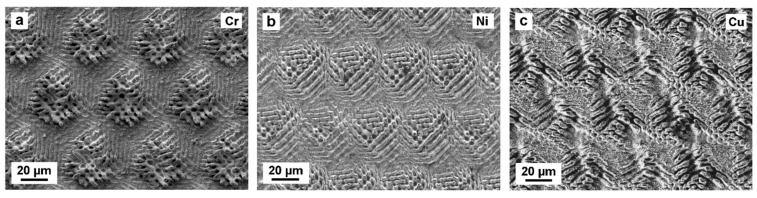
SEM images of (**a**) Cr, (**b**) Ni, and (**c**) Cu structured by four-beam DLIP with a fluence of 15.4 J/cm^2^ and 80 pulses.

Upon increasing the laser fluence up to 15.4 J/cm^2^, the pattern homogeneity in all three materials is drastically reduced, but the repetitive DLIP structures are still recognizable (see Figure 3). Since a large amount of molten material is redeposited around the interference minima positions, the cone-like structures tend to collapse giving place to large clusters, as in Figure 3a,b, and grooves, as shown in Figure 3b,c [55,56]. 

### 3.3. Effect of Pulse Number

**Figure 4:**
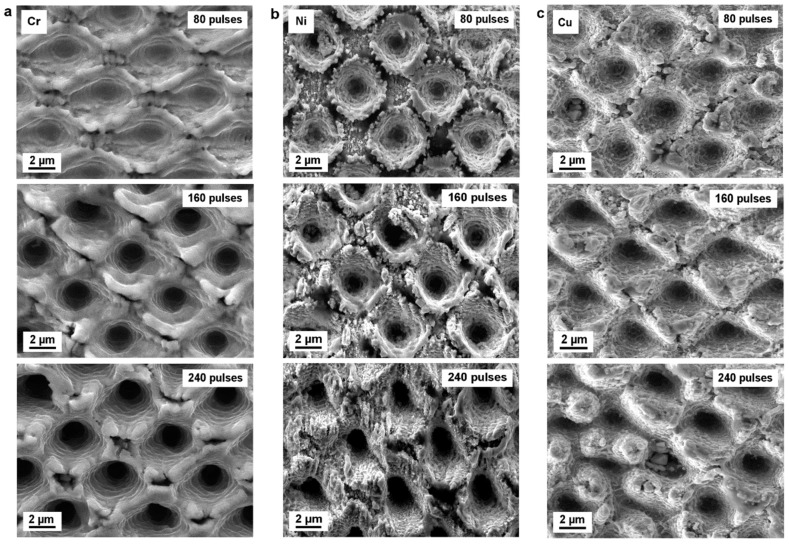
SEM images of (**a**) Cr, (**b**) Ni, (**c**) Cu substrates structured by direct laser interference patterning. The hole-like patterns were fabricated with a laser fluence of 2.9 J/cm^2^ and different numbers of laser pulses: 80, 160, and 240.

To study the effect of pulse number on the topography evolution, Cr, Ni, and Cu surfaces were irradiated with varying laser pulses, namely N = 1, 5, 10, 20, 40, 80, 100, 120, 160, 200, and 240, at a fixed laser fluence of 2.9 J/cm². The evolution of the surface morphology produced on the studied metals as a function of the number of applied pulses is shown in Figure 4. Using picosecond laser pulses, the DLIP process is dominated by the photothermal interaction between the laser radiation and the metal, which consists of local melting and/or selective ablation at the interference maxima positions [57,58,59]. The deep hole-like features shown in Figure 4 reveal the evident material removal at the interference maxima. The raised rims around those craters, which can be seen in all treated samples, were probably formed due to the redeposition of molten material around the holes [60,61]. It was also observed that a larger number of pulses correlates with deeper structures. As the number of laser pulses reaches 240, the surface structure becomes less defined, in particular for Ni and Cu, due to the redeposition of larger amount of material around the holes (see the bottom row in Figure 4b,c).

**Figure 5:**
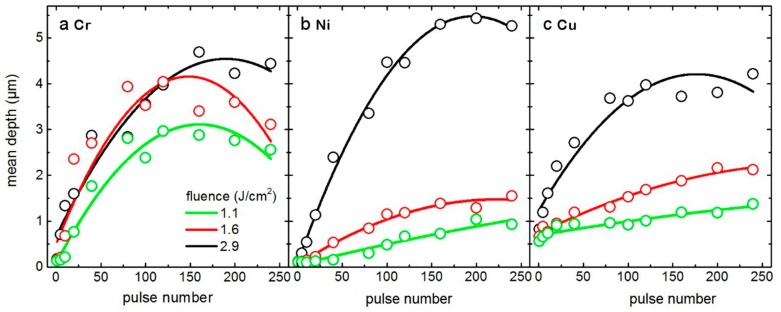
Dependence of the structure mean depth (open symbols) as a function of pulse number and with the fluence as a parameter on (**a**) Cr, (**b**) Ni, and (**c**) Cu. The lines are quadratic fits as a guide to the eye.

The structure depth of the produced periodic structures as a function of laser fluence and the number of pulses was measured using confocal optical microscopy and the results are shown in Figure 5. In general, the structure depth increases with both laser fluence and pulse number and it tends to saturate after ~120 pulses. A similar conclusion was also reported by Estevam-Alves et al. [62], who correlated this saturation effect to a decay of absorbed laser energy according to Lambert–Beer’s law. Although at a laser fluence of 2.9 J/cm^2^ the structure depth saturates to 4–5 µm in the three metals, at lower fluences the structure depth in Cr is significantly higher than in the other metals when more than 20 pulses are applied. This behavior could be attributed to differences in the ablation threshold fluence of the materials, since the material can only be selectively ablated at the interference maxima when the laser fluence is above the ablation threshold. According to [46], the theoretical ablation threshold fluence *F*_th_ can be calculated as:

(1) $$F_{th}=\rho L_{V}\sqrt{\alpha \cdot \tau_{p}},$$

(2) $$\alpha =K/\rho C_{p},$$

where ρ is the density of the metals, $L_{V}$ is the latent heat of vaporization, $\tau_{p}$ is the laser pulse duration, and α is the thermal diffusivity, which is related to the density, the thermal conductivity (K), and the heat capacity ($C_{p}$). Table 1 lists the values of some physical and thermal properties of the three metals used in this work. For 70 ps pulses, the calculated values for the ablation threshold fluence of Cr, Ni, and Cu are 0.20 J/cm^2^, 0.23 J/cm^2^, and 0.39 J/cm^2^, respectively. Since Cr has the lowest ablation threshold, it can be assumed a higher ablation rate on Cr at lower laser fluences, namely at 1.1 J/cm^2^–1.6 J/cm^2^, compared to Cu and Ni, which is consistent with the results observed in Figure 2 and Figure 5. In addition, reflectivity plays also a role in the laser ablation mechanism, since the lower the reflectivity, the higher the absorbed electromagnetic energy in the material. As seen in Table 1, Cr presents the lowest value of reflectivity of 0.55 which also contributes to the high ablation rate observed in Cr at low fluences.

The ablation threshold is not only a material dependent property, since it also strongly depends on the radiation conditions, for instance laser wavelength and pulse duration. Further experiments must be carried out in the future to determine the ablation threshold on these materials and its relationship with the behavior observed in Figure 5. Additionally, the multi-pulse ablation process involves different effects such as melting, recrystallization, quenching, native oxide layer formation involving exothermic processes, defect generation and phase transformation, that can result in unexpected structure depth dependences with the number of pulses and fluence as well as modified mechanical properties of the final structured surface [60,63,64].

### 3.4. LIPSS Formation and Evolution

LIPSS [65,66,67,68], a universal phenomenon that occurs after laser irradiation on a wide number of different materials [69,70,71], have been found to exhibit different characteristic shapes, including ripples (lines), rods, cones, grooves, etc. The generation of LIPSS takes place commonly only in a fluence range close to the material damage threshold and even just below the ablation threshold [72]. The total dose of laser energy, or accumulated fluence, influences the LIPSS formation as well as their morphology [72,73,74,75,76,77,78]. 

**Figure 6:**
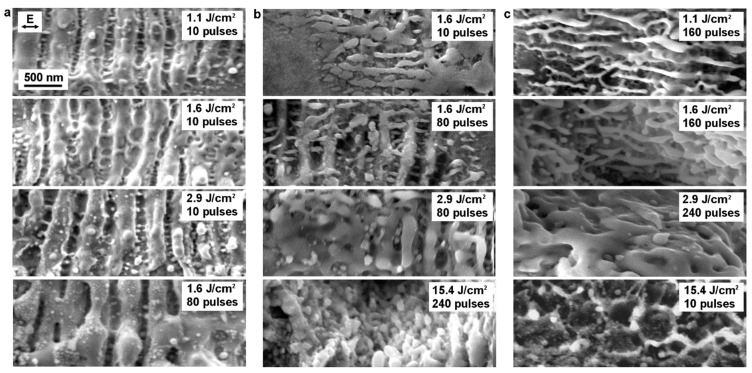
SEM images of irradiated (**a**) Cr, (**b**) Ni, and (**c**) Cu sheets by picosecond laser with varying fluence and pulse number. The arrow indicates the polarization direction of the laser pulses.

The sequence of SEM images in Figure 6 shows the evolution of LIPSS on Cr, Ni, and Cu sheets with varying energy dose. As shown in Figure 6a, nanoscaled periodic wave-like structures are produced on the Cr substrate, after irradiating with 10 laser pulses at a fluence of 1.1 J/cm^2^. The periodic ripples are oriented perpendicular to the laser polarization (double arrow in Figure 6a) and have an average spatial period of 410 nm, representing 77% of the laser wavelength. This type of LIPSS is commonly referred to as low spatial frequency LIPSS (LSFLs), which are usually assumed to be generated by the excitation of surface plasmon polaritons (SPP) [60,65,72,79,80,81]. Differently, smaller periodic structures, with a spatial period of approximately 120 nm, observed between the LSFLs are generally classified as high spatial frequency LIPSS (HSFL). This type of LIPSS was observed on several metals when applying ultra-short laser pulses with durations in the femtosecond and picosecond range [65,82]. Since the orientation of HSFLs is parallel to the laser polarization, they cannot be explained via SPP excitation [79,83]. Currently, the mechanism of HSFLs formation is still under debate [60,81,83]. 

Similar to the Cr structured surface, LIPSS are also observed on laser treated Ni, but only at higher irradiation doses. In Figure 6b, only HSFLs were induced on Ni at a fluence of 1.6 J/cm^2^ with 10 pulses, while LSFLs become visible only when the number of pulses increases to 80. The spatial period of LSFLs on Ni lies between 346 and 415 nm, and they also orient perpendicular to the irradiation polarization. The HSFLs have a spatial period between 129 and 207 nm and their orientation is parallel to the laser polarization. A different behaviour was observed on Cu, since no LSFLs are visible after irradiating the surface with laser fluences in the range between 1.1 J/cm^2^ and 15.4 J/cm^2^, whereas HSFLs are visible for 80 or more pulses and fluences between 1.1 J/cm^2^ and 1.6 J/cm^2^ (Figure 6c). The direction of the induced HSFLs on the Cu surface is also parallel to the polarization of the laser radiation, and the average spatial period was about 180 nm. 

In order to provide a systematic and qualitative overview of the observed LIPSS, a summary of the different types of LIPSS with various morphology, feature size and periodicity are listed in Table 2. The period of LSFLs on Cr and Ni are both slightly smaller than the laser wavelength, which is in accordance with previous results [65]. When the three metals are irradiated with a fluence higher than 2.9 J/cm^2^, the ripples started to melt and coalesce, vanishing from the surface. Subsequently, groove- and cone-like structures with larger feature sizes (Λ = 3.3–3.7 µm) appear on the surface of the three irradiated metals (see Figure 3). Differently, when the fluence is set to 15.4 J/cm^2^, grass-like nanostructures, with a feature size of approximately 90 nm, are visible only on Cu. Vorobyev et al. also reported the formation of these grass-like nanostructured LIPSS upon irradiating a Pt surface with 10 pulses at a fluence of 0.16 J/cm^2^ using an amplified Ti:sapphire laser system with a wavelength of 800 nm, and 65 fs pulse duration [68].

### 3.5. Hot Embossing

To test the molding performance of the DLIP structured metals, stamps with the same spatial period of 4.7 µm were used to imprint PMMA foils by hot embossing. The sidewall roughness of the stamps has to be as small as possible to prevent frictional forces between the embossing tool and the soften polymer become larger than the local tensile strength of the polymer [14]. For this reason, LIPSS formation inside the cavities should be reduced to minimize frictional forces and allow a smooth detachment of the polymer from the stamp without damaging the imprinted structures. To comply with this requirement, the metal stamps were structured after irradiating with 10 pulses at F = 2.9 J/cm^2^ (Cr), 120 pulses at F = 1.6 J/cm^2^ (Ni), and 80 pulses at F = 1.6 J/cm^2^ (Cu) yielding mean structure heights of 1.34 µm (Cr), 1.18 µm (Ni), and 1.31 µm (Cu), respectively. Although LIPSS cannot be completely suppressed applying these laser paramters, LSFLs and HSFLs are only formed in the areas between the cavities of the Cr stamp as seen in Figure 2a (bottom row). In turn, in the cavities of the Ni and Cu molds only HSFLs with a period ranging from 150 nm to 200 nm are present.

**Figure 7:**
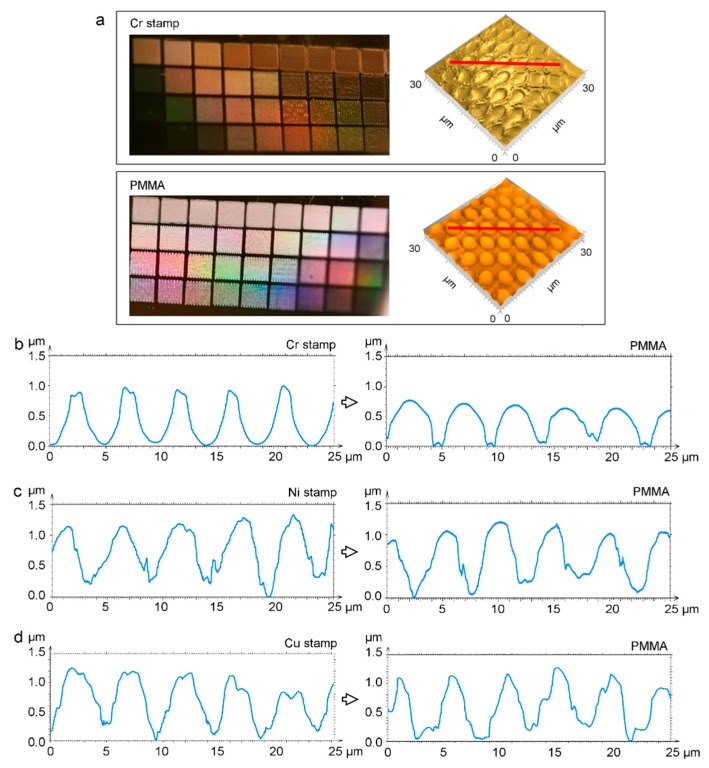
(**a**) Photographs and 3D topographies of a (top) Cr stamp and (bottom) embossed PMMA substrate. The red lines correspond to the positions where the profiles in (**b**) were extracted. (b, c, d) Topographical profiles of laser-treated metals (**b**) Cr, (**c**) Ni, (**d**) Cu, and the corresponding PMMA substrates imprinted from them by hot embossing. The embossing temperature was 100 ± 5 °C, the applied force was 200 kN, and the embossing time was 5 min.

The three-dimensional confocal microscope images in Figure 7a confirmed the successful replication of the periodic pattern from the Cr mold to the PMMA substrate. The imprint parameters, i.e., temperature: 100 °C, applied force: 200 kN and imprint time: 5 min, were optimized to allow the soften PMMA polymer to fill the holes of the mold. The produced hemispherical microlenses present smooth surfaces and a suitable homogeneity. The average diameter of the microlenses is 3.6 µm with a standard deviation of 0.4 µm, whereas the spatial period is 4.7 µm. To gain a deeper insight into the imprint quality, topographical profiles of the three master molds and the corresponding imprinted polymers were extracted as shown in Figure 7b–d. It can be seen in Figure 7b, that the cavities sidewalls of the Cr stamp are smooth. While the mean structure depth of the Cr master was 1.34 µm, the sag height of the imprinted convex microlenses was 1.12 µm, indicating that the plastic material did not completely fill the stamp cavities. This is probably due to trapped air between polymer and cavity [12]. Figure 7c shows that the polymer embossed with the Ni stamp has a low homogeneity as well as insufficient quality. This can be explained by the high surface roughness visible at the sidewalls in the hole-like structures. In this case, the microlens height is 1.18 µm matching the mold structure depth, suggesting that in contrast to the Cr mold, the trapped air could flow outwards through the voids along the crater rims in the mold. However, the imprinted microlenses on PMMA have a smaller diameter of 3.3 µm compared to that of the micro-holes on the Ni stamp (3.6 µm), which may be attributed to the incomplete mold filling due to the surface roughness of the mold sidewalls. Similar conclusions can be drawn from the imprinted foils employing the Cu stamp as shown in Figure 7d, where the average diameter of the embossed microstructures is only 2.9 µm (compared to 3.5 on Cu stamp), and the average height is 1.27 µm. 

## 4. Conclusions

In this work, a comparison study was performed to assess the capability of direct laser interference patterning to produce Cr, Ni, and Cu molds for nanoimprinting systems. Using pulsed ps-laser radiation with optimized processing parameters, hole-like micropatterns with satisfactory quality and homogeneity were produced on those metal substrates. It was found that the pattern topography and the evolution of LIPSS are strongly dependent on the laser fluence and the number of applied pulses. For instance, LSFLs were found in Cr and Ni with their orientation perpendicular to the laser polarization and with a period slightly smaller than the laser wavelength. In turn, HSFLs were identified on the three metals showing spatial periods between 100 nm and 200 nm and an orientation parallel to the radiation polarization. The structure depth in the three materials tends to increase as the number of laser pulses increases to 120, followed by a saturation around a constant depth. The maximum achieved structure depth in these three metals was between 4 and 5 µm after applying ~160 pulses at a fluence of 2.9 J/cm^2^. 

The laser induced microstructures on the three metals were replicated on PMMA foils using a hot embossing process. When Cr was used as mold material, imprint the results showed a reliable reproduction of homogenously distributed hemispherical microlenses with a mean diameter and depth of 3.6 µm and 1.12 µm, respectively. In contrast, the PMMA substrates embossed with the Ni and Cu stamps showed a low quality and a relatively small microlens diameter caused by the rough cavities’ sidewalls of these stamps. Further studies will be conducted to optimize the laser and imprint parameters to structure large areas as well as curved molds for roll-to-roll hot embossing systems.

## Figures and Tables

**Table 1:** Parameters of untreated metal sheets used in experiments [46]. The reflectivity was calculated from the optical constants of the metals according to [47,48,49].

Parameter	Cr	Ni	Cu
Thickness (µm)	315	266.67	101.25
Surface roughness $R_{a}\;$(µm)	0.05	0.05	0.17
Density ρ (g/cm^3^)	7.1	8.9	8.96
Latent heat of vaporization $L_{V}$ (J/g)	6580	6378	4796
Thermal conductivity K (W/(m∙K))	93.7	91	401
Heat capacity $C_{p}$ (J/(g∙K))	0.52	0.44	0.39
Diffusivity α (cm^2^/s)	0.25	0.23	1.16
Reflectivity (@532 nm)	0.55	0.63	0.6

**Table 2:** Summary of types, orientation relative to radiation polarization, and period of LIPSS on irradiated Cr, Ni, and Cu surfaces with varying laser fluence and number of pulses.

Material	LIPSS Type	Orientation Relative to Polarization	Mean Period (µm)	Mean Diameter (µm)	Fluence (J/cm2)	Pulse Number
**Cr**	LSFLs	perpendicular	0.41	-	1.1–2.9	10–80
	HSFLs	parallel	0.12	-	1.1–2.9	10–80
	Grooves	parallel	3.6	-	15.4	80–240
	Cone-like	-	-	2.3	15.4	80–240
**Ni**	LSFLs	perpendicular	0.38	-	1.6–2.9	80
	HSFLs	parallel	0.17	-	1.1–2.9	10–240
	Grooves	parallel	3.7	-	15.4	80–160
	Cone-like	-	-	1.9	15.4	80–240
**Cu**	HSFLs	parallel	0.18	-	1.1–1.6	80–240
	Grass-like	-	-	0.09	15.4	10–80
	Grooves	parallel	3.3	-	15.4	80
	Cone-like	-	-	3.6	15.4	160–240
